# 103. Expansion of an Antimicrobial Stewardship Program Through Implementation of a Discharge Verification Queue

**DOI:** 10.1093/ofid/ofab466.305

**Published:** 2021-12-04

**Authors:** Ellen C Rubin, Alison L Blackman, Eleanor K Broadbent, David Wang, Ilda Plasari, Pawlose Ketema, Karrine Brade, Tamar F Barlam

**Affiliations:** Boston Medical Center, South Boston, Massachusetts

## Abstract

**Background:**

Antimicrobial stewardship programs (ASPs) have traditionally focused interventions on inpatient care to improve antibiotic prescribing. Support of effective interventions for ASPs targeting antibiotic prescriptions at hospital discharge is emerging. Our objective was to expand stewardship services into the outpatient setting through implementation of a process by the antimicrobial stewardship team (AST) to verify antimicrobials prescribed at discharge.

**Methods:**

This quality improvement initiative incorporated a discharge order verification queue managed by AST pharmacists to review electronically prescribed antimicrobials Monday through Friday, from 8:00 am to 4:00 pm. The queue was piloted Sep 2020 and expanded hospital-wide Feb 2021. Patients < 18 years old and those with observation or emergency department status were excluded. The AST pharmacist reviewed discharge prescriptions for appropriateness, intervened directly with prescribers, and either rejected or verified prescriptions prior to transmission to outpatient pharmacies. Complicated cases were reviewed with the AST physician to evaluate intervention appropriateness. Interventions were categorized as either dose adjustment, duration, escalation or de-escalation, discontinuation, or safety monitoring.

**Results:**

A total of 602 prescriptions were reviewed between Sep 2020 and Apr 2021. An AST pharmacist intervened on 28% (171/602) of prescriptions. The most common intervention types were duration (41%, 70/171), discontinuation (18%, 31/171), and dose adjustment (17%, 30/171). The most common indications in which the duration was shortened was community acquired pneumonia (26%, 18/70), skin and soft tissue infection (21%, 15/70), and urinary tract infection (17%, 12/70). The most common antibiotics recommended for discontinuation were cephalexin (32%, 10/31) and trimethoprim-sulfamethoxazole (10%, 3/31). The overall intervention acceptance rate was 78%.

**Conclusion:**

An AST pharmacist review of antimicrobial prescriptions at discharge improved appropriate prescribing. The discharge queue serves as an effective stewardship strategy for inpatient ASPs to expand into the outpatient setting.

**Disclosures:**

**All Authors**: No reported disclosures

